# Association between type of feeding at hospital discharge and nutritional status of Brazilian very preterm infants: a multicenter study

**DOI:** 10.1016/j.jped.2024.06.006

**Published:** 2024-07-15

**Authors:** Betina Soldateli, Rita C. Silveira, Renato S. Procianoy, Erika M. Edwards, Mandy B. Belfort

**Affiliations:** a Faculdade de Medicina, Departamento de Nutrição, Universidade Federal do Rio Grande do Sul (UFRGS), Porto Alegre, RS, Brazil; b Serviço de Nutrição e Dietética, Hospital de Clinicas de Porto Alegre (HCPA), Porto Alegre, RS, Brazil; c Faculdade de Medicina, Departamento de Pediatria, Universidade Federal do Rio Grande do Sul (UFRGS), Porto Alegre, RS, Brazil; d Neonatologia, Hospital de Clínicas de Porto Alegre (HCPA), Porto Alegre, RS, Brazil; e Vermont Oxford Network, Burlington, VT, USA; f College of Engineering and Mathematical Sciences, University of Vermont, Burlington, VT, USA; g The Robert Larner, M.D. College of Medicine, University of Vermont, Burlington, VT, USA; h Department of Pediatrics, Brigham and Women's Hospital, Boston, MA, USA

**Keywords:** Very-low-birth-weight infant, Human milk, Infant nutrition, Growth, Neonatal intensive care unit

## Abstract

**Objectives:**

To examine trends over time in diet and size of very preterm infants, and associations of diet with size at hospital discharge/transfer.

**Methods:**

The authors studied 4062 surviving very preterm infants born < 32 weeks’ gestational age and < 1500 g between January 2012 and December 2020 from 12 Brazilian Neonatal Intensive Care Units. Diet type at discharge/transfer was classified as exclusive human milk, exclusive formula, or mixed. Outcomes were weight and head circumference at hospital discharge and the change in each from birth to discharge. The authors used linear regression to estimate adjusted associations of diet type with infant size, overall, and stratified by fetal growth category (small vs. appropriate for gestational age). The authors also examined trends in diet and infant size at discharge over the years.

**Results:**

Infants’ mean gestational age at birth was 29.3 weeks, and the mean birth weight was 1136 g. Diet at discharge/transfer was exclusive human milk for 22 %, mixed for 62 %, and exclusive formula for 16 %. Infant size in weight and head circumference were substantially below the growth chart reference for all diets. Infants fed human milk and mixed diets were lighter and had smaller heads at discharge/transfer than infants fed formula only (weight z: −2.0, −1.8, and −1.5; head z: −1.3, −1.2 and −1.1 for exclusive human milk, mixed and exclusive formula respectively).

**Conclusion:**

Results suggest high human milk use but gaps in nutrient delivery among hospitalized Brazilian very preterm infants, with little evidence of improvement over time.

## Introduction

Neuro-nutrition is a key concept for brain health where each nutrient has its specific function in the growth and development of the brain of preterm infants, reinforcing the role of the motherʼs milk as a nutritional gold standard.^1^ Despite human milk being the recommended diet, alone it is insufficient to support optimal postnatal growth among very preterm (VPT) infants (<32 weeks) during hospitalization because dietary requirements for protein and other nutrients are substantially higher than for full-term infants.^2^ Hospitalized VPT preterm infants are therefore uniquely vulnerable to undernutrition. Although many factors influence brain development, the NICU diet is important since it is modifiable within clinical care.^3^

Preterm births (< 37 weeks of gestation) represent 10 % of births in Brazil,^4^ and a few reports provide information about NICU diet and growth outcomes in this population. In terms of diet, from 2010 to 2019, the number of newborns who received human milk in Brazilian NICUs increased by 35 %, with a 29 % increase in the volume of human milk.^5^ In the U.S., the use of human milk increased modestly over a similar time span.^6^ Regarding growth outcomes, a study from 2007 to 2011 showed that 26 % of infants experienced growth faltering for weight and 5 % for head circumference.^7^ In United States NICUs, growth outcomes have improved markedly in the past two decades, but the extent to which Brazil has experienced similar improvements is poorly described.^6^^,^^7^

NICU diet and infant growth are related. In Brazil, one study tested the association between the type of feeding at hospital discharge and the mean z-score differences in weight and head circumference of 649 very low birth weight (VLBW) infants, born between 2006 and 2013, with no differences in these anthropometric measurements in comparative analyses.^8^ A ten-year study of VPT infants born <32 weeks of gestation from 777 United States NICUs showed improvements in growth across all discharge diet types (human milk only, mixed, formula only),^9^ but notably, infants receiving human milk only (no formula or fortifier) at discharge had experienced slower weight gain and head growth than infants receiving formula or mixed diets. In another cohort of over 1400 hospitalized VLBW infants from 9 NICUs in Massachusetts, the authors found similar growth outcomes among infants fed formula as compared with fortified human milk, but among human milk-fed infants, those who received predominantly donor milk had poorer growth than those fed predominantly or exclusively maternal milk.^10^ Overall, these findings suggest that feeding unfortified human milk and feeding a predominantly donor milk diet, may place VPT infants at risk for impaired growth during a critical period for brain development.

Given the strong emphasis on human milk in Brazilian NICUs, understanding the relationships between feeding and growth in this context is essential to drive improvements in care and ultimately long-term neurodevelopmental outcomes.^11^ The aims of this study were to:1) examine trends over time in Brazilian very preterm infants’ diet at discharge; 2) examine trends over time in infant size at discharge; and 3) examine associations of very preterm infant diet with size at discharge/transfer.

## Methods

This cross-sectional study used data from Vermont Oxford Network (VON), a voluntary worldwide community dedicated to improving the quality, safety, and value of newborn care through a coordinated program of data-driven quality improvement, education, and research.^12^ Among the Brazilian centers participating in VON, 12 NICUs agreed to provide their data for this study; they were from different regions of Brazil, seven from public hospitals, and five from private. Each center collected the data according to the definitions and guidelines of the VON Manual of Operations.^13^

The authors included all surviving VPT (< 32 weeks gestational age) and VLBW (< 1500 g) infants from 12 Brazilian NICUs, born from January 1, 2012 to December 31, 2020. From 4811 infants the authors excluded those discharged > 42 weeks of gestation (*n* = 388); discharged/transferred < 15 days (*n* = 26); with congenital anomalies (*n* = 164); missing diet (*n* = 137) or covariate data (*n* = 34). Implausible z-scores likely to error (> 4 <) were excluded, but not the individuals; therefore, the total sample may differ slightly.

The authors categorized the diet at NICU discharge/transfer as 1) exclusive human milk (no formula or fortifier); 2) mixed diet (human milk with fortifier or formula); and 3) exclusive formula. Exclusive human milk was considered when the infant received human milk directly from the breast or expressed. A mixed diet was classified as receiving formula and/or human milk supplemented with formula or fortifier. The formula could be standard, special, or for premature infants. All enteral diets were considered, regardless of whether they were directly from the breast, bottle, cup, feeding tube, gastrostomy, or other method.^13^

Infant size measures included weight and head circumference at birth and discharge; the authors calculate z-scores at birth and at discharge/transfer - which reflects infant size relative to Fentonʼs preterm growth charts^14^; average weekly rate of head growth – cm/week (HC at discharge minus HC at birth/length of stay); and weight change from birth to discharge in grams/kg/day was assessed by the two-point exponential model.^15^

### Data analysis

The authors used linear regression models to estimate marginal means for infant size/growth in each parameter (weight and head z score at discharge, weight change, head growth), adjusting for potential confounders and clustering within hospitals with generalized estimating equations (GEE). The authors tested all assumptions of linearity between exposure and outcome and continuous variables included in the adjusted model (gestational age at birth, birth weight z score, postmenstrual age at discharge, sex of the infant, multiple gestation, and NICU morbidity – necrotizing enterocolitis and chronic lung disease). The authors compared the adjusted means among three diet categories (exclusive human milk, mixed, exclusive formula) and ran models stratified by fetal growth category (small vs. appropriate). Considering the Zika virus outbreak in Brazil in 2016 as the cause of microcephaly,^16^ the authors ran a sensitivity analysis for head circumference excluding this year. To describe infant size outcomes over time the authors used GEE, testing the slope by year, with no adjustment in mean variables. Analyses were performed in SPSS 29 and graphs using the means over the years in R v4.2.0 software (package ggplot2).

### Ethics

The proposal was submitted by VON to the responsible neonatologist for each center with a request for authorization to use the data from each site. By the time of the request, Brazil had 43 centers registered in the VON network; among these, 12 authorized the use of the data without patient or center identification. The project was registered in Plataforma Brazil and approved by the Ethics Committee of Hospital de Clinicas under the number 2020-0333 and CAAE number 33598020.5.0000.5327.

## Results

We included data from 4062 VPT infants from 12 Brazilian NICUs from 2012 to 2020 in the study. From these, 903 infants (22 %) were receiving an exclusive human milk diet, 652 (16 %) exclusive formula, and 2507 (62 %) mixed diet at discharge/transfer. The formula-fed infants were a week younger in gestational age and had over 100 g lower birth weights. Table 1 shows the sample characteristics overall and by diet group.

**Table 1:** Characteristics of the study population overall and by diet type at discharge (*n* = 4062).

**Characteristics**	Overall (4062)	HM (903)	Mixed (2507)	Formula (652)
Male, n (%)	1944 (48.0 %)	399 (44.2 %)	1209 (48.2 %)	336 (51.5 %)
Singleton, n (%)	3171 (77.9 %)	780 (86.4 %)	1883 (75.1 %)	508 (77.9 %)
C-section, n (%)	2803 (68.8 %)	528 (58.5 %)	1886 (75.2 %)	389 (59.7 %)
NEC, n (%)	126 (3.1 %)	19 (2.1 %)	75 (3.0 %)	32 (4.9 %)
Late-onset sepsis, n (%)	929 (23.1 %)	167 (18.5 %)	524 (20.9 %)	238 (36.5 %)
Chronic lung disease, n (%)	619 (15.1 %)	62 (6.9 %)	405 (16.2 %)	152 (23.3 %)
**At birth**				
Inborn, n (%)	3890 (95.7 %)	853 (94.5 %)	2420 (96.5 %)	617 (94.6 %)
Gestational age, weeks, mean (SD)	29.3 (1.8)	29.7 (1.7)	29.3 (1.8)	28.7 (1.9)
Weight. grams, mean (SD)	1136 (230)	1193 (218)	1132 (230)	1077 (229)
Weight z score, mean (SD)	−0.4 (0.8)	−0.4 (0.8)	−0.5 (0.8)	−0.4 (0.8)
HC. cm, mean (SD)	26.5 (2.0)	26.8 (2.0)	26.5 (2.0)	26.1 (2.1)
HC z score, mean (SD)	−0.2 (1.1)	−0.2 (1.1)	−0.2 (1.0)	−0.1 (1.1)
SGA, n (%)	610 (15 %)	135 (15.0 %)	375 (15.0 %)	100 (15.3 %)
**At discharge or transfer**				
Postmenstrual age, weeks, mean (SD)	37.3 (2.1)	36.4 (1.9)	37.4 (2.0)	38.3 (2.3)
Weight, grams, mean (SD)	2203 (453)	1953 (342)	2231 (422)	2442 (536)
Weight z score, mean (SD)	−1.9 (0.9)	−2.1 (0.9)	−1.9 (0.9)	−1.8 (1.0)
HC, cm, mean (SD)	32.0 (1.9)	31.2 (1.7)	32.1 (1.9)	32.6 (2.2)
HC z score, mean (SD)	−1.1 (1.0)	−1.2 (0.9)	−1.0 (1.0)	−1.1 (1.1)
Length of stay, days median (P25; P75)	52 (40; 69)	43 (34;58)	53 (41; 69)	65 (50;82)

Mixed diet indicates human milk plus formula or fortifier; SGA= small for gestational age (<10th percentile); z-scores according Fenton charts; HM=human milk; NEC= enterocolitis necrotizes; HC= head circumference;.

SD, standard deviation; P25, percentile 25; P75, percentile 75.

Table 2 shows the overall mean size and growth parameters in each diet group and adjusted differences between groups. Weight change was fastest in the exclusive formula and mixed-diet groups, and head growth was fastest similarly in exclusive formula and mixed-diet groups, compared with exclusive human milk. Weight and head z-score at discharge/transfer deviated less from the growth reference in the exclusive formula group. Also, shows the results stratified by gestational age category (small vs appropriate). Among small for gestational age (SGA) infants, absolute weight change was faster than inappropriate (AGA) infants (e.g., for formula-fed infants, weight change was 15.3 g/kg/d in SGA infants compared with 13.0 g/kg/d in AGA infants). Weight and head z score at discharge deviated more from the growth reference in the SGA group compared with the AGA. Otherwise, head growth was similar in both SGA and AGA infants. The authors also performed a sensitivity analysis limited to the head outcome, excluding the year 2016 due to the Zika virus outbreak in Brazil, with no differences in the results (data not shown).

**Table 2:** Diet type at discharge, weight change, head growth, and infant size 2012–2020, total sample and stratified by infant size for gestational age (*n* = 4062).

		Adjusted mean ± SE			Difference (95 % CI)	
Growth measure	All*	(1) Exclusive HM	(2) Mixed diet	(3) Exclusive Formula	(1) vs. (2)	(2) vs. (3)
	*n* = 4062	*n* = 876	*n* = 2439	*n* = 625		
Weight z-score	−1.9 (0.9)	−2.0 (0.1)	−1.8 (0.1)	−1.5 (0.1)	**−0.2 (−0.3, −0.06)**	**−0.3 (−0.4, −0.2)**
Weight change, g/kg/d	11.8 (3.2)	11.2 (0.5)	12.3 (0.4)	13.3 (0.5)	**−1.0 (−1.4, −0.7)**	**−1.0 (−1.3, −0.8)**
Head z-score	−1.1 (1.0)	−1.3 (0.1)	−1.2 (0.1)	−1.1 (0.1)	**−0.1 (−0.2, −0.04)**	**−0.1 (−0.2, −0.03)**
Head growth, cm/week	0.7 (0.3)	0.64 (0.04)	0.68 (0.04)	0.70 (0.05)	**−0.04 (−0.06, −0.01)**	−0.02 (−0.05, 0.009)

***Small for gestational age***						

***n=610***						
Weight z-score	−2.9 (0.6)	−2.8 (0.1)	−2.6 (0.1)	−2.3 (0.1)	**−0.2 (−0.3, −0.1)**	**−0.3 (−0.4, −0.1)**
Weight change, g/kg/d	13.5 (3.1)	12.9 (0.4)	14.1 (0.5)	15.3 (0.5)	**−1.2 (−1.9, −0.6)**	**−1.2 (−1.8, −0.5)**
Head z-score	−1.7 (0.9)	−1.9 (0.2)	−1.6 (0.2)	−1.7 (0.2)	**−0.3 (−0.4, −0.1)**	0.04 (−0.2, 0.2)
Head growth, cm/week	0.7 (0.3)	0.61 (0.03)	0.71 (0.03)	0.69 (0.03)	**−0.1 (−0.2, −0.005)**	0.02 (−0.03, 0.08)

***Appropriate for gestational age***						

***n=3452***						
Weight z-score	−1.8 (0.9)	−1.9 (0.1)	−1.7 (0.1)	−1.4 (0.1)	**−0.2 (−0.3, −0.06)**	**−0.3 (−0.4, −0.2)**
Weight change, g/kg/d	11.5 (3.1)	11.0 (0.6)	12.1 (0.5)	13.0 (0.5)	**−1.0 (−1.4, −0.7)**	**−1.0 (−1.2, −0.7)**
Head z-score	−1.0 (1.0)	−1.2 (0.1)	−1.1 (0.1)	−1.0 (0.09)	**−0.1 (−0.2, −0.02)**	**−0.1 (−0.2, −0.03)**
Head growth, cm/week	0.7 (0.3)	0.65 (0.05)	0.68 (0.05)	0.70 (0.05)	**−0.03 (−0.05, −0.01)**	−0.03 (−0.06, 0.003)

Weight and head z-score at discharge/transfer according to Fenton charts. Human milk indicates unfortified human milk. A mixed diet indicates human milk plus formula or fortifier. Linear regression estimates adjusted for sex, gestational age at birth, birth weight z-score, postmenstrual age at discharge, multiple gestation, neonatal intensive care unit morbidity, and hospital. Differences are statistically significant (95 % CI excludes 0). Small for gestational age (<10th percentile).

⁎ All show unadjusted results (mean±SE).

**Figure 1:**
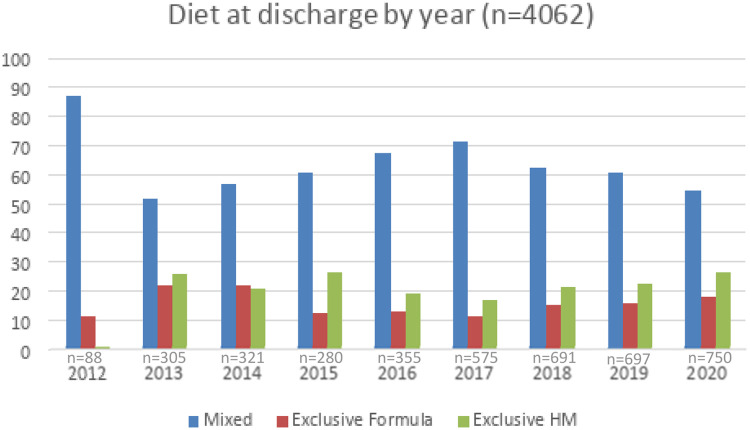
Trends over time in VLBW/VPT infant diet at discharge or transfer (*n* = 4062).

**Figure 2:**
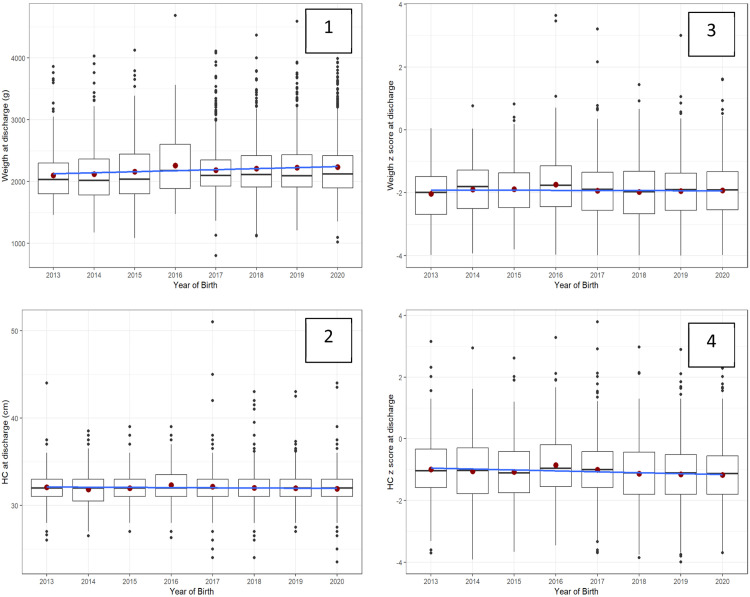
Trends in infant size at discharge by year of birth (2013–2020) in all diets (*n* = 3974). *Red circle=mean; black line= median; blue line=slope; 1-weight (grams), 2-head (cm), 3-weight and 4-head z-scores. ** Mean, median, and slope in 1, 2, 3, and 4 graphs show a lack of improving change trends over time.

Figure 1 shows similar trends over time in VLBW/VPT infant diet at discharge or transfer, except for the year 2012. Figure 2 shows trends in infant size at discharge by year of birth (2013–2020) in all diets. Data from 2012 was excluded from the analysis (*n* = 88). For each year weight at discharge increased by a mean of 10 g, and no differences over the years were demonstrated in the weight z score at discharge. For each year head circumference at discharge decreased by a mean of −0.04 cm, and the head z-score at discharge decreased by a mean of −0.03 standard deviation. In this period, size outcomes deviated from the growth chart reference.

## Discussion

This data analysis from 12 NICUs in Brazil had three main findings. One, 22 % of infants received a non-fortified exclusive human milk diet at discharge and 62 % received some human milk (mixed diet). Two, weight and head circumference at discharge/transfer were well below the Fenton reference, without evidence of improvement in these growth outcomes from 2012 to 2020. Three, infants receiving a non-fortified exclusive human milk diet deviated more from the growth reference compared to infants receiving other diets, especially those infants born SGA.

First, human milk is the recommended diet in the NICU because it provides nutrients and bioactive factors that are essential for VPT/VLBW infant physical growth, protection from complications such as NEC, and neurodevelopment (1, 3). In the present study, the most prevalent feeding type at discharge in Brazilian NICUs was the mixed diet (66 %), which includes any combination of human milk, formula, and/or fortifier; and 22 % of infants received an unfortified exclusive human milk diet at discharge. Overall, 84 % received at least some human milk at NICU discharge. Most prior studies reporting diet at discharge were smaller than ours and results from some are difficult to compare due to differences in diet definitions. Three single-center studies reported diet at discharge during a time period similar to the present study. One was conducted in a private NICU in southern Brazil and found that 77 % (*n* = 259) of VLBWs were receiving a mixed diet and 16 % (*n* = 54) were receiving exclusive mother's milk at discharge.^17^ Overall, 93 % were receiving at least some human milk, which is higher than in the present study. In a public hospital in Rio de Janeiro, 11 % of VLBW infants were exclusively breastfeeding at hospital discharge.^18^ And another in Sao Paolo, including 649 VLBW infants, 40 % received a mixed diet at discharge, and 10 % received an exclusive human milk diet, with overall human milk feeding (50 %) lower than in this study.^8^ Moreover, a report including 1438 VLBW from 23 to 33 weeks gestational age from 20 NICUs from public hospitals showed 29 % of infants on an exclusive human milk diet at discharge in 2015.^19^ In South America, a pooled analysis with 406,309 VLBW infants admitted to 1113 NICUs from 2009 to 2016 reported 85 % of infants in mixed diet and 14 % in exclusive human milk at discharge.^20^ In most of the Brazilian studies, exclusive human milk at discharge was higher than in a U.S. study of over 275,000 infants from 777 NICUs in which 50 % received any human milk and 6.6 % of infants received exclusive human milk at discharge/transfer.^9^ The high percentage of infants receiving human milk at NICU discharge in Brazil can be explained in part by the substantial efforts in the use of human milk in the Brazilian NICUs.^21^ Also, differences in human milk at discharge between the U.S. and Brazil may be explained by policies, healthcare systems, and culture.

Second, the present findings of infant discharge size outcomes indicate clinically significant growth faltering relative to a fetal reference, without evidence of improvement from 2012 to 2020. A longitudinal study using VON data from 4 NICUs in the city of Rio de Janeiro from 2007 to 2011 analyzed growth and size outcomes at discharge from 570 VLBW. The 4 NICUs reported similar and standardized clinical and nutritional practices with the use of fortified motherʼs milk or preterm formula in the absence of mother's milk. The mean z-score of weight at discharge was −1.54 ± 0.75, and for head circumference was −0.45 ± 0.94; indicating better outcomes than the present study (z-scores of −1.9 ± 0.9 for weight and −1.1 ± 1.0 for head circumference). One possible explanation for the difference is the standardized fortification practices reported in the 4 NICUs.^7^ These findings are important since the analysis pointed out, in a preceding period of this study, growth faltering in a VLBW preterm infant sample similar to ours. The present study adds since including information from 2012 to 2020 on size outcomes, that it did not improve over the years, besides reporting diet data analysis at discharge. These findings contrast starkly with a U.S. study including 824 VON member hospitals with 314,811 infants born 24 to 29 weeks’ gestational age at birth, between 2005 and 2018, which reported increases in discharge weight within all birth gestational ages and across all NICU types. During that time, the proportion of infants discharged home from the hospital on human milk increased.^6^

Third, in the diet and growth analysis, the present findings showed that infants receiving an exclusive human milk diet deviated more from the growth reference compared to other diets (weight z: −2.0, −1.8, and −1.5; head z: −1.3, −1.2 and −1.1 for exclusive human milk, mixed and exclusive formula, respectively). One single-center study in Brazil tested the association between the type of feeding at discharge and the z-score change in weight and head circumference of 649 very low birth weight infants, born between January 2006 and December 2013. The z-score change for exclusive human milk, mixed, and exclusive formula groups were: –0.84, –1.02, and –0.86 for weight; and –0.21, –0.52, and –0.08 for head circumference, with no differences in these anthropometric measurements in comparative analyses. The study also reported a significant difference between the formula and mixed groups in the adjusted z-score model for length (8). However, the study differs in some aspects of ours since the authors did not report length and demonstrated infant size at discharge adjusted for birth size, rather than absolute z-score change. Moreover, findings are limited considering the exclusive human milk group was small (10 % of all). In a US analysis of 777 NICU with VON data, Belfort et al. found similarly that z-score change for weight deviated more from the fetal reference for infants fed both human milk diets compared with formula only (−0,88; −0.82 and −0.80 for human milk-only, mixed and formula only respectively) and also for head z-score change (−0.52, −0,49, −0,45). Otherwise, a ten-year trend in growth by diet groups showed improvements in growth likely reflecting improving nutritional care.^9^ These differences may reflect fortification practices in these different settings.

In Brazil, human milk, including the mother's own milk, is routinely pasteurized before feeding to hospitalized VPT/VLBW infants. This is relevant because human milk processing, including pasteurization, alters the milk's composition. Findings of a systematic review suggest the potential for two-fold and greater differences in the fat, protein, and energy composition of donor milk compared with raw human milk, with mean values for energy and fat often below clinical reference values expected for human milk, and even fortified donor milk frequently does not reach preterm recommendations for some nutrients.^22^ Evidence regarding differences in preterm infant growth outcomes based on maternal versus donor milk analysis is conflicting and still inconclusive.^10^^,^^23^ However, a predominant donor milk diet, despite fortification, may contribute to infants’ undernutrition during NICU stay.^24^ From this perspective, the finding of poor infant growth/size in the exclusive unfortified human milk diet, besides lack of fortification, may be also explained by the substantial use of pasteurized human milk during hospitalization in the Brazilian NICUs.

The infant size trends at discharge/transfer indicate that the physical growth of Brazilian very preterm infants in the NICU deviated from the reference fetus over the years, with no improvement, suggesting reduced nutrient accretion during a critical period for brain development. For decades Brazil has had a strong policy to incentivize and improve human milk use in the NICUs,^25^ however, little has been done to improve national fortification practices. It is important considering that Brazilian VPT infants are receiving the benefits of a human milk diet; however, the human milk diet is highly variable in its composition and requires fortification to meet the unique needs of preterm infants and prevent growth restriction.^26^ Despite the human milk diet has been a common practice, fortification must be encouraged in NICU settings to promote adequate infant growth in this vulnerable population.^27^

Strengths of this study include large sample size and being the first to analyze diet and growth trends at hospital discharge in VLBW Brazilian preterm infants from 12 NICUs. This study had limitations, such as the cross-sectional design, describing feeding and size at only one-time point. Moreover, there is a potential for minimizing differences due to confounding by reverse causation, since infants considered growing poorly may have been put on some formula supplements for discharge^28^; also, confounding by unmeasured clinical and/or social factors, despite covariate adjustment. Still, anthropometric indicators such as weight and head circumference are used as proxies for nutrient accretion, and head circumference is only a crude proxy for brain size.^3^

Finally, these findings can encourage health teams to optimize feeding practices – especially fortification of human milk - to improve the growth outcomes of these infants in Brazil.

## Conclusion

An important percentage of VTP/VLBW infants still receive an unfortified human milk diet in the NICU during a crucial period for brain development. Diet and infant size trends over time have not improved in Brazil. VPT/VLBW infants classified as SGA at birth despite the weight gain and head growth being similar to AGA during hospitalization, had presented poor infant size at discharge.

## Funding

This study received financial support from the FIPE - Hospital de Clínicas de Porto Alegre.

## Conflicts of interest

The authors declare no conflicts of interest.
